# ElemCor: accurate data analysis and enrichment calculation for high-resolution LC-MS stable isotope labeling experiments

**DOI:** 10.1186/s12859-019-2669-9

**Published:** 2019-02-19

**Authors:** Di Du, Lin Tan, Yumeng Wang, Bo Peng, John N. Weinstein, Fredric E. Wondisford, Xiaoyang Su, Philip L. Lorenzi

**Affiliations:** 1 Department of Bioinformatics and Computational Biology, The University of Texas MD Anderson Cancer Center, Houston, TX USA; 2 Department of Medicine, Division of Endocrinology, Robert Wood Johnson Medical School, Rutgers University, New Brunswick, NJ USA; 3 Cancer Institute of New Jersey, Rutgers University, New Brunswick, NJ USA

**Keywords:** LC-MS, Stable isotope labeling, High resolution, Data correction

## Abstract

**Background:**

The investigation of intracellular metabolism is the mainstay in the biotechnology and physiology settings. Intracellular metabolic rates are commonly evaluated using labeling pattern of the identified metabolites obtained from stable isotope labeling experiments. The labeling pattern or mass distribution vector describes the fractional abundances of all isotopologs with different masses as a result of isotopic labeling, which are typically resolved using mass spectrometry. Because naturally occurring isotopes and isotopic impurity also contribute to measured signals, the measured patterns must be corrected to obtain the labeling patterns. Since contaminant isotopologs with the same nominal mass can be resolved using modern mass spectrometers with high mass resolution, the correction process should be resolution dependent.

**Results:**

Here we present a software tool, ElemCor, to perform correction of such data in a resolution-dependent manner. The tool is based on mass difference theory (MDT) and information from unlabeled samples (ULS) to account for resolution effects. MDT is a mathematical theory and only requires chemical formulae to perform correction. ULS is semi-empirical and requires additional measurement of isotopologs from unlabeled samples. We validate both methods and show their improvement in accuracy and comprehensiveness over existing methods using simulated data and experimental data from *Saccharomyces cerevisiae*. The tool is available at https://github.com/4dsoftware/elemcor.

**Conclusions:**

We present a software tool based on two methods, MDT and ULS, to correct LC-MS data from isotopic labeling experiments for natural abundance and isotopic impurity. We recommend MDT for low-mass compounds for cost efficiency in experiments, and ULS for high-mass compounds with relatively large spectral inaccuracy that can be tracked by unlabeled standards.

## Background

Stable isotope labeling experiments have been increasingly popular in quantitative, targeted metabolomics [1–4]. Metabolite isotopologs that are labeled differently can be distinguished by mass spectrometry. The resolved mass distribution vectors (MDV) for all possible mass isotopologs of individual metabolites are independent of metabolite levels and correspond to the degree of isotopic tracer labeling [4]. With tracer analysis and metabolic flux analysis, MDV provides quantitative information on pathway activity and pathway contribution variation [5]. Because naturally occurring isotopes [6] and tracer isotopic impurity from the nutrient [5, 7, 8] contribute to the measured signal, the fractional abundance of measured isotopologs (FAM) collected from the instrument must be corrected to obtain MDV.

Existing correction methods are typically based on a correction matrix constructed by calculating theoretical contribution from isotopic natural abundance of each element and isotopic impurity of the tracer element using combinatorics [9]. Such calculations work well on low resolution instruments. However, modern mass spectrometers with high resolving power can easily resolve isotopologs with the same nominal mass, and, thus, including all isotopologs in the correction matrix is no longer justified. To address that limitation, fractional abundances of metabolites measured from an unlabeled sample can be used to construct the correction matrix [10]. The resolution effect can also be theoretically incorporated using mass difference theory based on nominal instrument resolution and exact mass differences between isotopologs from different chemical elements [8]. Nevertheless, the existing implementations of both methods have mathematical defects.

Here we present ElemCor with correction and improvement of those two methods and a user-friendly graphical interface. We validate both methods using simulations and experiments, and we show their improvements upon other existing methods introduced above. We also discuss the strength of the two different methods and suggest applications to different types of studies.

## Results

### Correction for simulated data

**Fig. 1:**
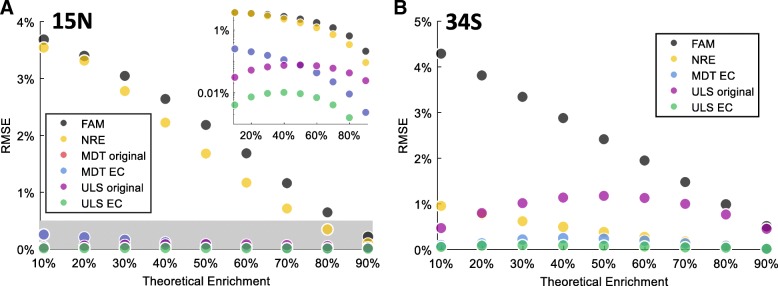
**a** Root mean square error (RMSE) of ^15^N enrichments from simulated data of 24 metabolites after correction using different methods. The gray region corresponds to the mean standard deviation in ^15^N experiments shown later in Fig. 2. The inset shows the same figure in logarithmic scale. EC stands for ElemCor. **b** RMSE of ^34^S enrichments from simulated data of 10 metabolites after correction using different methods. Nominal instrument resolution for both (**a**) and (**b**) is 140,000

We first simulated FAM for 24 ^15^N enriched small metabolites including ADP, ATP, CTP, GDP, N-acetyl-glutamate, N-acetyl-glutamine, N-carbamoyl-L-aspartate, UDP, UDP-D-glucose, UDP-N-acetyl-glucosamine, UTP, arginine, asparagine, citrulline, glutamate, glutamine, glutathione, glutathione disulfide, lysine, ornithine, phenylalanine, serine, tryptophan, and uridine. Different correction methods were used to obtain MDV and calculate isotopic enrichment, which was compared to theoretical value. Root mean square errors (RMSEs) between the isotopic enrichments obtained for all 24 metabolites and their theoretical value (20%, See Methods and Materials) were calculated for all methods. For all 24 metabolites considered and all degrees of theoretical enrichment, MDT and ULS resulted in significantly lower RMSE from theoretical enrichment than FAM, correction without considering resolution effect (NRE, directly using the correction matrix defined in Theoretical Correction Matrix section), and even the mean standard deviation of experimental results (Fig. 1a).

A detailed comparison between different MDT and ULS methods is shown in the inset of Fig. 1a. The modified ULS implemented in ElemCor yielded significantly more accurate results than the other three methods. The original ULS yielded accuracy similar to that of the two MDT methods, which are both theoretical and do not incorporate additional experiment measurements. Although the difference between the modified MDT in ElemCor and original MDT is mathematically significant, in practice, the modified MDT in ElemCor did not yield noticeably different results (< 0.1%) than the original MDT, as shown by the overlapped blue and red markers. That is because the difference between them is the balanced combination of all isotopes (Fig. 5b), which has very small numerical contribution to the correction matrix during the calculation of multinomial probabilities. The contribution may increase as the molecular weight of a metabolite increases.

We then simulated FAM for ten ^34^S enriched small metabolites including thiamine pyrophosphate, glutathione disulfide, S-adensyl-methione, cystathione, cystine, glutathione, cysteine, thiamine, taurine, and methionine (Fig. 1b). The original MDT did not include correction for ^34^S tracer and, thus, was not evaluated here. Similarly, for all ten metabolites considered and all degrees of theoretical enrichment, the modified MDT and ULS in ElemCor were remarkably more accurate than NRE and FAM. The original ULS, however, yielded lower accuracy than NRE, indicating that the resolution effect was not properly modeled. The reason of ULS being inaccurate in ^34^S simulation is that the most abundant isotopes of sulfur has much less fractional abundance than that of nitrogen (95.0% ^32^S vs. 99.6% ^14^N). Therefore, deconvolution of fractional natural abundance of sulfur from the column vector will make a significant difference in the diagonal of the correction matrix.

**Fig. 2:**
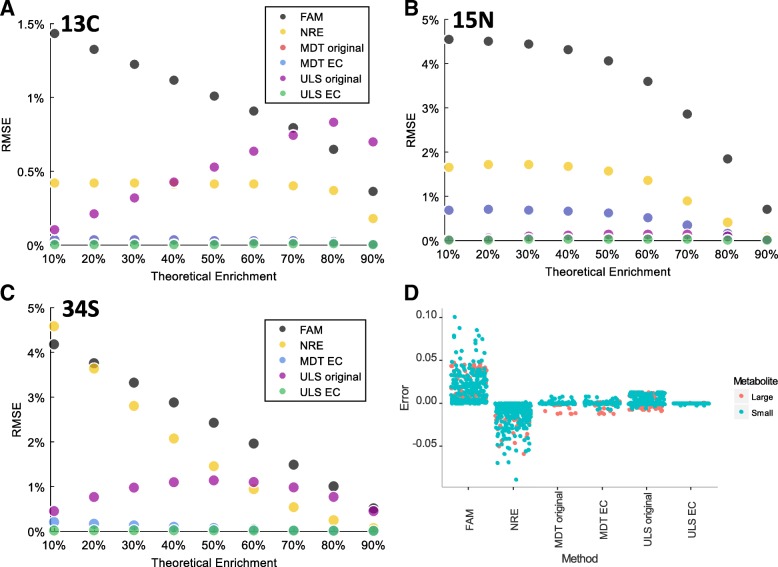
Root mean square error (RMSE) of (**a**) ^13^C, (**b**) ^15^N, and (**c**) ^34^S enrichments from simulated data of four CoA metabolites after correction using different methods. EC stands for ElemCor. Nominal instrument resolution for (**a**), (**b**), and (**c**) are 280,000. **d** The errors of correction for all simulated metabolites under all degrees of theoretical enrichments

We also evaluated the accuracy of correction for larger metabolites, with a focus on coenzyme A (Fig. 2a-c). The metabolites considered include coenzyme A (CoA), acetyl-CoA, succinyl CoA, and HMG-CoA. RMSEs between the isotopic enrichments obtained for those 4 metabolites and their theoretical value 20% were calculated for all methods. For all tracers considered, the modified MDT and ULS methods in ElemCor yielded accurate corrected results, as indicated by lower RMSE, whereas the accuracy of NRE was generally not acceptable and was even worse than FAM for ^34^S at 10% theoretical enrichment. The accuracy of the original ULS was excellent for ^15^N but was remarkably lower for ^13^C and ^34^S, which were accurately calculated by ElemCor. Similarly, the modification of MDT did not make a noticeable numerical change. Note that for large metabolites, higher instrument resolution is typically used. Therefore, we used 280,000 in our simulation.

Figure 2d shows the errors of correction from different methods for all simulated data. The error in FAM before correction is up to 10%. The modified ULS in ElemCor was most accurate, while the modified MDT in ElemCor was in the second place with slight lower accuracy. Although the original MDT did not differ noticeably from the modified MDT, the tracer element is limited to ^13^C, ^2^H, and ^15^N. The original ULS generally undercorrected the data with an error up to 1.5%. Correction without considering the resolution effect mostly overcorrected the data with an error up to 10%. Taken together, these results demonstrate that the resolution effect can contribute significantly when correction for natural abundance is performed. The two methods used in ElemCor outperform the other methods in accuracy.

### Correction for experiment data

**Fig. 3:**
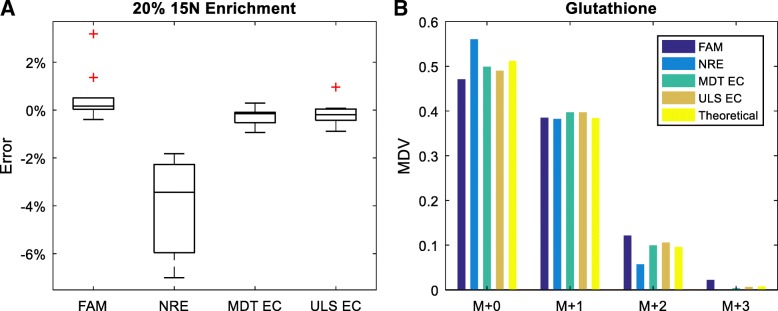
**a** Error in the measured ^15^N enrichment of ten metabolites after correction using different methods. The red crosses represent outliers outside interquartile range. **b** MDV of glutathione after correction using different methods. EC stands for ElemCor

We also performed a yeast experiment to validate ElemCor. ^15^N was chosen as the tracer element due to the independent incorporation of tracer atoms, allowing the binomial calculation of theoretical MDV. Not all metabolites studied in simulation have measurable enrichment from natural abundance in unlabeled samples, and therefore only ten metabolites were considered in the experiments. For all ten metabolites considered, MDT and ULS yielded more accurate results than FAM and NRE (Fig. 3a). The slight advantage of ULS over MDT shown in simulation was not present in experiments because the standard deviation of experimental measurements was larger than the advantage itself. The enrichment of one metabolite, glutathione, was not properly corrected by ULS; Fig. 3b illustrates that ULS yielded a slight undercorrection for glutathione, which is likely due to inaccurate measurement of the unlabeled samples near the limit of detection.

### Software

**Fig. 4:**
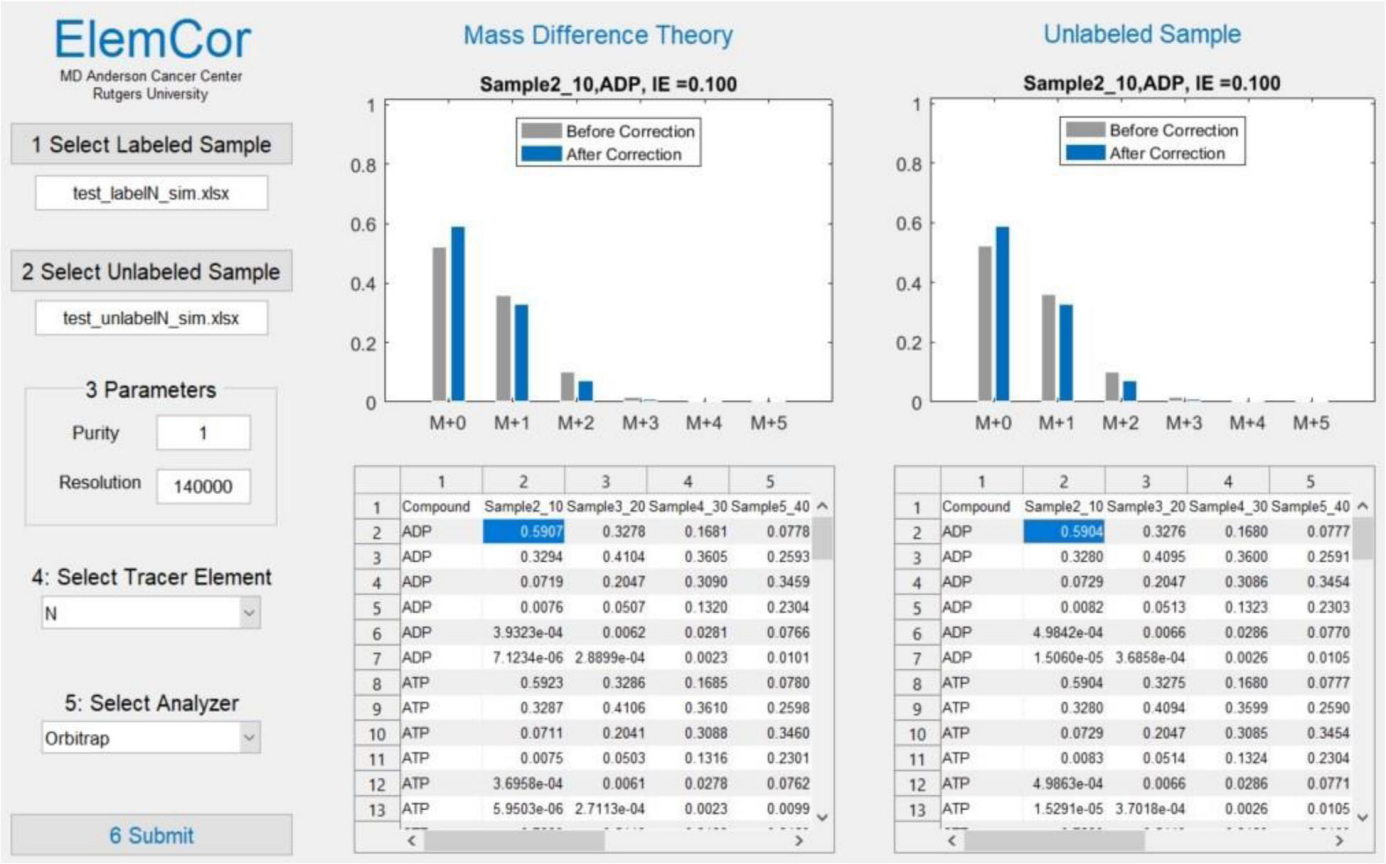
User interface of ElemCor

ElemCor has a friendly user interface that guides users through six easy steps (Fig. 4). In Steps 1 and 2, labeled and unlabeled data (*.xlsx) are loaded. Step 2 is optional, and when it is not performed, ElemCor runs based on MDT only. In Steps 3 to 5, isotopic purity of the tracer, nominal instrument resolution, tracer element, and the type of mass analyzer are specified. In addition to ^13^C, ^2^H, and ^15^N, ElemCor allows ^18^O and ^34^S as the tracer element for correction. Finally, in Step 6 the loaded data are analyzed, and isotopic enrichment is calculated for each compound. When a user selects a cell in the data table, MDV and FAM for the corresponding compound and sample are shown in the figure window. The results are automatically saved in separated sheets in the original file.

ElemCor can be used to correct and analyze data in both tracer analysis and metabolic flux analysis. For tracer analysis where direct comparison of isotopic enrichments is needed, the GUI can accurately and rapidly perform such analysis without requiring a programming background. For metabolic flux analysis where MDVs will be used to calculate pathway fluxes, since popular flux analysis software suites such as FiatFlux [11] and INCA [12] are also MATLAB-based, the provided MATLAB function of ElemCor can easily be adapted to those large-scale workflows.

## Discussion

MDT is a mathematical theory and requires only chemical formulae to perform correction. ULS, on the other hand, is a semi-empirical method that incorporates the measurement of unlabeled samples into the correction matrix. It does not require chemical formulae but requires additional experimental measurement of unlabeled samples. They have previously been used to perform corrections for isotopic labeling experiments with defective implementations [8, 10]. The software tool ElemCor provides correction for those mathematical defects and performance improvement for those two methods. Compared to the original ULS method in Ref. [10], the improved ULS method used in ElemCor is significantly more accurate and removes negative MDV thanks to non-negative regression. The improved MDT used in ElemCor is as accurate as the original MDT method in Ref. [8], but it extends the correction algorithms to support more tracer elements and mass analyzer types. In addition to those performance improvements, ElemCor also provides a user-friendly graphical interface that enables easy data import and direct visualization of the correction process and corrected MDV.

MDT and ULS are both sufficiently accurate to perform correction for natural abundance. In our simulated data tests, ULS was slightly more accurate than MDT across all compounds. In our experimental data tests, MDT was marginally more accurate for only specific compounds. MDT is sufficiently accurate for low-mass metabolites, and the additional accuracy provided by ULS may be overshadowed by experiment error. Considering the cost of additional experiment measurement, we recommend using MDT for small metabolites. We generally recommend using ULS for large metabolites, since the accuracy of MDT is noticeably lower (Fig. 2b and d). Moreover, the unlabeled samples can also track instrument bias and provide correction for instrument spectral discrepancy which becomes significant for large metabolites [8]. If heavier labeled fractions are under-measured due to instrument bias, ULS will use the distorted, unlabeled FAM as the input and therefore has a better chance of achieving a more accurate enrichment calculation.

## Conclusion

We present here a software tool, ElemCor, that corrects LC-MS data from isotopic labeling experiments for natural abundance and isotopic impurity. ElemCor uses two methods—mass difference theory (MDT) and unlabeled samples (ULS) —to account for the resolution effect. We demonstrate that ElemCor corrects the mathematical errors found in previously published methods, and includes more options for tracer elements and analyzers than previously published methods.

We used simulated data with enrichment in different tracer atoms to evaluate MDT and ULS. For all compounds considered, correction without considering resolution effect (NRE, used in IsoCor) was significantly less accurate than other correction methods and not noticeably more accurate than directly using the uncorrected fractional abundances of measured isotopologs (FAM). Those findings confirm that the resolution effect needs to be considered during correction. The modified ULS method used in ElemCor is more accurate than the original ULS method and is, in fact, the most accurate of all methods tested. The modified MDT used in ElemCor was not noticeably more accurate than the original MDT. Nevertheless, the modified MDT improves upon the limitation of tracer elements and analyzer type by including two more tracer elements (oxygen and sulfur) and one more analyzer (FTICR).

In summary, considering the significant cost of experiments, we recommend MDT for low-mass compounds, where the additional accuracy provided by ULS is barely noticeable and may be overshadowed by experiment errors. For high-mass compounds, we recommend ULS. Additional inclusion of an unlabeled standard can track instrument bias, which is important for large metabolites with relatively large spectral inaccuracy. Overall, ElemCor addresses the limitations of previous stable isotope correction methods and facilitates accurate correction of mass spectrometry-based stable isotope tracer data.

## Methods

### Theoretical correction matrix

The correction is essentially a linear regression defined by the total correction matrix C,

1 $$ {Cx}^{\prime}\kern0.5em =\kern0.5em z $$

where *z* (*z*_0_, *z*_1_, *z*_2_,  … , *z*_*N*_)^*T*^ includes the fractional abundances of measured ions (FAM) and *x* = *x*^′^/|*x*^′^|_1_ (*x*_0_, *x*_1_, *x*_2_,  … , *x*_*N*_)^*T*^ is the MDV with the contribution of natural abundance and isotopic impurity removed from FAM [5]. Here T stands for transpose and |*x*|_1_ stands for sum or L1-norm of *x*. The sum of *z* is 1 by definition. Since *C* typically has a norm less than 1, the sum of *x*^′^ is usually larger than 1. The constraints include: 1) the sum of *x* is 1; and 2) all components of *x* are non-negative. The total correction matrix *C* is the product of: i) the individual correction matrices for natural abundance of non-tracer elements; ii) the correction matrix for natural abundance of the tracer element; and iii) the correction matrix for isotopic impurity of the tracer element. Note that matrix multiplication is not commutative, and the order of multiplication should not be changed from the one given above.

The correction matrix for a non-tracer element Q is expressed as

2 $$ {C}_1\kern0.5em =\kern0.5em \left(\begin{array}{ccccc}{q}_{0,{N}_q}& 0& 0& \cdots & 0\\ {}{q}_{1,{N}_q}& {q}_{0,{N}_q}& 0& \cdots & 0\\ {}{q}_{2,{N}_q}& {q}_{1,{N}_q}& {q}_{0,{N}_q}& \cdots & 0\\ {}\vdots & \vdots & \vdots & \ddots & \vdots \\ {}{q}_{N_t,{N}_q}& {q}_{N_t-1,{N}_q}& {q}_{N_t-2,{N}_q}& \cdots & {q}_{0,{N}_q}\end{array}\right) $$

Here ${q}_{i,{N}_q}$ (*i* = 0, 1, 2, … , *N*_*t*_) are the isotopolog natural abundance of Q where *N*_*q*_ is the number of mass isotopologs for Q excluding base mass and *N*_*t*_ is the number of mass isotopologs for a tracer element T excluding base mass. Note that *q*_*i*, *j* < *i*_ = 0 if *N*_*t*_ > *N*_*q*_. Only the isotopolog abundances at the lowest *N*_*t*_ + 1 masses are likely to be detectable and included in MDV, and thus matrix *C*_1_ is typically truncated with *N*_*t*_ + 1 rows remaining, yielding a square matrix [11–13]. The correction matrix for the tracer element T is expressed as

3 $$ {C}_2\kern0.5em =\kern0.5em \left(\begin{array}{ccccc}{p}_{0,{N}_t}& 0& 0& \cdots & 0\\ {}{p}_{1,{N}_t}& {p}_{0,{N}_t-1}& 0& \cdots & 0\\ {}{p}_{2,{N}_t}& {p}_{1,{N}_t-1}& {p}_{0,{N}_q}& \cdots & 0\\ {}\vdots & \vdots & \vdots & \ddots & \vdots \\ {}{p}_{N_t,{N}_t}& {p}_{N_t-1,{N}_t-1}& {p}_{N_t-2,{N}_t-2}& \cdots & {p}_{0,0}\end{array}\right) $$

Here *p*_*i*, *j*_ (*i* = 0, 1, 2, … , *N*_*t*_) are the isotopolog natural abundance of T where *N*_*t*_ is the number of isotopologs for T excluding base mass. *p*_*i*, *j*_ are the probabilities of finding +*i* mass due to natural abundance in the remaining *j* positions of tracer atoms [6].

The isotopolog natural abundance of element T (or Q) can be calculated using combinatorics. When there are two stable isotopes for T (e.g., ^12^C/^13^C) with natural abundance 1 − *α* and α respectively, and the total number of T atoms in the molecule is equal to *j*, *C*_2_ can be expressed explicitly with ${p}_{i,j}={C}_j^i{\left(1-\alpha \right)}^{j-i}{\alpha}^i$. When there are more than two stable isotopes (e.g., ^16^O/^17^O/^18^O), *C*_2_ has to be obtained numerically through multinomial distribution or iterative convolution [8, 9].

The correction matrix for isotopic impurity of the tracer is expressed as

4 $$ {C}_3\kern0.5em =\kern0.5em \left(\begin{array}{ccccc}{r}_{0,0}& {r}_{0,1}& {r}_{0,2}& \cdots & {r}_{0,{N}_t}\\ {}0& {r}_{1,1}& {r}_{1,2}& \cdots & {r}_{1,{N}_t}\\ {}0& 0& {r}_{2,2}& \cdots & {r}_{2,{N}_t}\\ {}\vdots & \vdots & \vdots & \ddots & \vdots \\ {}0& 0& 0& \cdots & {r}_{N_t,{N}_t}\end{array}\right) $$

Here *r*_*i*, *j*_ (*i* = 0, 1, 2, … , *N*_t_) are the probabilities of finding the *i*^*th*^ isotopolog when the nutrient has isotopic impurity given that the *j*^*th*^ (*j* = *i*, *i* + 1, … , *N*_t_) isotopolog is found when the nutrient is pure. When there are two stable isotopes for T, ${r}_{i,j}={C}_j^i{\beta}^{j-i}{\left(1-\beta \right)}^i$, where *β* is the impurity of the tracer element. Similarly, when there are more than two stable isotopes, *r*_*i*, *j*_ can be obtained numerically using multinomial distribution or iterative convolution. Note that isotopic purities are reported at the atomic level. For example, U-^13^C_6_ glucose with 99% isotopic purity has 94% glucose with all carbons labeled by ^13^C. The number of individual correction matrices to be included is dependent on the chemical formula of the compound of interest. For example, if a compound has one tracer element and two non-tracer elements, the total correction matrix is then $C={C}_1{C}_1^{\prime }{C}_2{C}_3$, where *C*_1_ and ${C}_1^{\prime }$ correspond to the individual correction matrices for the two non-tracer elements, respectively.

### Unlabeled samples (ULS)

The aforementioned formulation fails to consider resolution of the isotopologs and, therefore, may be inaccurate for high-resolution instruments. To address that limitation, measured fractional abundances of the compound in an unlabeled sample can be used to approximate the effect of resolution on the correction matrix [10]. Theoretically, metabolites from an unlabeled sample have no isotopic enrichment in MDV, namely *x* = (1, 0,  … , 0)^*T*^. Therefore, according to Eq. (1), the FAM from an unlabeled sample corresponds to the first column of the correction matrix for natural abundance. However, that vector should not be used as-is to construct every column of the correction matrix, as done by others [10].

**Fig. 5:**
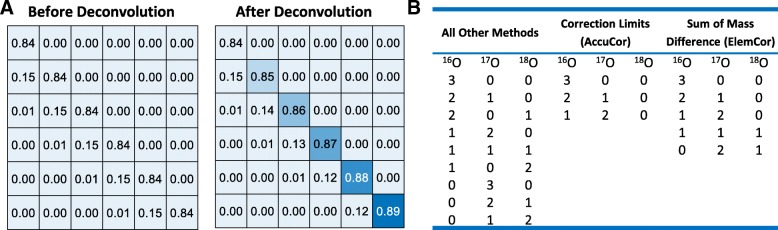
**a** Comparison of the correction matrices for natural abundance before and after deconvolution of acetyl-CoA using the ULS method. FAM from labeled samples is from Ref. [10]. The left panel shows the correction matrix used in Ref. [10], and the right panel shows the correction matrix used in ElemCor. Matrices are truncated to show the first six rows and columns for illustration purposes. **b** Combinations of oxygen atoms included in the correction matrix for different methods. The example is for glutamine at an instrument resolution of 100,000. Note that the total mass excess over base mass due to the heavy isotopes of oxygen should not exceed the number of tracer (carbon) atoms in the molecule

In fact, the column vectors in the correction matrix are different since the number of tracer atoms considered for natural abundance is different for every column (Eq. 3) [6, 9]. It has been shown by researchers that convolution is an efficient way to construct the column vectors of the correction matrix [9]. This is because the *N*th order multinomial coefficients are components of a vector obtained by *N* convolutions of the event probability vector. Since the number of tracer atoms considered for natural abundance is different by one for neighboring columns [6], besides the padded zeros, the difference between two neighboring columns in the correction matrix for natural abundance is simply a convolution of the fractional natural abundance of the tracer element. As a result, one can obtain the correction matrix for natural abundance by deconvolution of the fractional natural abundance of the tracer element *N*_*t*_ times from the FAM from an unlabeled sample. Figure 5(a) shows the effect of deconvolution on the correction matrix for natural abundance for acetyl-CoA, which was used as an example in [10]. Deconvolution remarkably changes the components on the main diagonal as indicated by their colors.

Since unlabeled samples do not contain any information about the labeling agent, the FAM from an unlabeled sample only helps to construct the correction matrix for natural abundance. The correction matrix for isotopic impurity of the tracer needs to be constructed using Eq. (4). We implemented those corrections in ElemCor.

### Mass difference theory (MDT)

Mass difference theory (MDT) can also be used to zero out certain components of the correction matrix based on the actual instrument mass resolution [8]. A non-tracer-labeled ion can be resolved from the tracer-labeled ion and excluded from the correction matrix if the mass (m/z) difference satisfies $\Delta  M\ge 1.66{M}^{1.5}/R\sqrt{M_R}$ or *∆M* ≥ 1.66*M*^2^/*RM*_*R*_ for Orbitrap or FTICR analyzers. Here *∆M* is the mass difference between the two ions, *M* is accurate mass of the tracer-labeled isotopolog, *R* is nominal instrument resolution, and *M*_*R*_ is the *m/z* where the nominal instrument resolution is defined and is classically 200 for Orbitrap and 400 for FTICR [8, 14]. This criterion is used to calculate the correction limit for each non-tracer heavy isotope. For example, the smallest resolvable mass difference, *∆M*, for glutamine (C_5_H_10_N_2_O_3_) isotopologs under a nominal resolution of 100,000 in Orbitraps is 2.07 ∙ 10^−3^. The mass difference between a ^17^O_1_-glutamine ion and a labeled ^13^C_1_-glutamine ion is 8.62 ∙ 10^−4^. Therefore, the correction limit of ^17^O is the nearest integer less than or equal to 2.07 ∙ 10^−3^/8.62 ∙ 10^−4^, and is equal to 2. That is, only two ^17^O atoms can be “disguised” as ^13^C atoms and need to be considered in the correction matrix. Similarly, the correction limits of all the M + 1 heavy isotopes such as ^15^N, and ^2^H are both zero (mass differences of 6.32 ∙ 10^−3^ and 2.92 ∙ 10^−3^ respectively), yielding both of them resolvable under the resolution. For M + 2 heavy isotope ^18^O, the mass difference between an ^18^O_1_-glutamine ion and a labeled ^13^C_2_-glutamine ion is 2.46 ∙ 10^−3^ > 2.07 ∙ 10^−3^, and thus it has a correction limit of zero as well. As a result, the original MDT only considers ^13^C and two ^17^O atoms in the correction matrix for glutamine at a resolution of 100,000.

The determination of isotope exclusion in the correction matrix based on correction limits of individual isotopes is not self-consistent. For example, for glutamine at a resolution of 100,000, the correction limits for ^17^O and ^18^O are 2 and 0, respectively, indicating that ^18^O is resolvable and, therefore, excluded. However, due to the opposite signs of the two mass differences, a non-tracer labeled ion with two ^17^O atoms and one ^18^O atom has a mass difference of 7.39 ∙ 10^−4^ (< the smallest resolvable mass difference 2.07 ∙ 10^−3^) from the corresponding labeled ion and should actually be included in the correction matrix. The weakness of the correction limits used in the original MDT can be circumvented by directly calculating the sum of mass differences from all isotopes for determination. Figure 5(b) illustrates the combinations of different oxygen atoms of glutamine considered in the correction matrix for different methods.

### Implementation

We developed a software tool, ElemCor, for correction of stable isotope tracer data using both ULS and MDT to construct the correction matrix and a non-negative constraint in the regression. ElemCor is a stand-alone application with a friendly graphical interface. Non-negative linear regression of Eq. (1) followed by normalization to the sum of 1 is used to obtain MDV in ElemCor, and isotopic enrichment is calculated as ${\sum}_{i=1}^Ni\cdot {x}_i$ [9, 15]. The correction matrix in Eq. (1) was constructed using MDT and/or ULS as described above. ElemCor was developed under MATLAB (2016b) environment. An ElemCor function without graphical interface is also available so it can be easily adapted to most metabolic flux analysis software suites, which are also MATLAB-based [16, 17].

### Validation datasets

We used both simulated and experimental data to validate ElemCor. Simulations at incremented nutrient enrichments were performed using the isotope simulation module in Xcalibur (Thermo Fisher Scientific). The chemical formula describing the isotopolog mixture for 20% ^15^N glutamine (C5H10N2O3 × 0.64 + C5H10[15]NNO3 × 0.32 + C5H10[15]N2O3 × 0.04) and resolutions of 140,000 and 280,000 were used for the simulation. Experiments were performed on yeast (*S. cerevisiae*) grown in 1% glucose and yeast nitrogen base (0.5% NH_4_Cl) with 20.0% ^15^N in ammonium for labeled samples and 0% for unlabeled samples [8]. The isotopic purity of the nutrient is 99%. Each sample was harvested from 4 mL of yeast cell culture when the OD_600_ reached 0.6. Cell extract was used in the LC-MS analysis (Orbitrap Q Exactive PLUS Mass Spectrometer, Thermo Fisher Scientific).

## Abbreviations

FAM: Fractional abundance of measured isotopologs
MDT: Mass difference theory
MDV: Mass distribution vector
ULS: Unlabeled samples

## References

[1] Jiang L, Shestov AA, Swain P, Yang C, Parker SJ, Wang QA, Terada LS, Adams ND, McCabe MT, Pietrak B (2016). Reductive carboxylation supports redox homeostasis during anchorage-independent growth. Nature.

[2] Martínez-Reyes I, Diebold Lauren P, Kong H, Schieber M, Huang H, Hensley Christopher T, Mehta Manan M, Wang T, Santos Janine H, Woychik R (2016). TCA cycle and mitochondrial membrane potential are necessary for diverse biological functions. Mol Cell.

[3] Guan D, Xiong Y, Borck PC, Jang C, Doulias P-T, Papazyan R, Fang B, Jiang C, Zhang Y, Briggs ER: Diet-induced circadian enhancer remodeling synchronizes opposing hepatic lipid metabolic processes. Cell 2018, 174(4):831–842. e812. 10.1016/j.cell.2018.06.031 PMC6086765 30057115

[4] Jang C, Chen L, Rabinowitz JD (2018). Metabolomics and isotope tracing. Cell.

[5] Buescher JM, Antoniewicz MR, Boros LG, Burgess SC, Brunengraber H, Clish CB, DeBerardinis RJ, Feron O, Frezza C, Ghesquiere B (2015). A roadmap for interpreting 13C metabolite labeling patterns from cells. Curr Opin Biotechnol.

[6] Lee WN, Byerley LO, Bergner EA, Edmond J (1991). Mass isotopomer analysis: theoretical and practical considerations. Biol Mass Spectrom.

[7] Shlomi T, Fan J, Tang B, Kruger WD, Rabinowitz JD (2014). Quantitation of cellular metabolic fluxes of methionine. Anal Chem.

[8] Su X, Lu W, Rabinowitz JD. Metabolite spectral accuracy on Orbitraps. Anal Chem. 2017;89(11):5940-5948. 10.1021/acs.analchem.7b00396 PMC5748891 28471646

[9] Millard P, Letisse F, Sokol S, Portais JC (2012). IsoCor: correcting MS data in isotope labeling experiments. Bioinformatics.

[10] Trefely S, Ashwell P, Snyder NW (2016). FluxFix: automatic isotopologue normalization for metabolic tracer analysis. BMC Bioinformatics.

[11] Chinkes DL, Aarsland A, Rosenblatt J, Wolfe RR. Comparison of mass isotopomer dilution methods used to compute VLDL production in vivo. Am. J. Physiol. 1996;271(2 Pt 1):373-383. 10.1152/ajpendo.1996.271.2.E373 8770033

[12] Van Winden W, Wittmann C, Heinzle E, Heijnen J. Correcting mass isotopomer distributions for naturally occurring isotopes. Biotechnol. Bioeng. 2002;80(4):477-479. 10.1002/bit.10393 12325156

[13] Wittmann C, Heinzle E. Mass spectrometry for metabolic flux analysis. Biotechnol. Bioeng. 1999;62(6):739-750. 10.1002/(sici)1097-0290(19990320)62:6<739::aid-bit13>3.0.co;2-e 10099575

[14] Zubarev RA, Makarov A. Orbitrap Mass Spectrometry. Anal. Chem. 2013;85(11):5288-5296. 10.1021/ac4001223 23590404

[15] Chevrier S, Crowell HL, Zanotelli VR, Engler S, Robinson MD, Bodenmiller B. Compensation of Signal Spillover in Suspension and Imaging Mass Cytometry. Cell Syst. 2018;6(5):612-620. 10.1016/j.cels.2018.02.010 PMC5981006 29605184

[16] Zamboni N, Fendt S-m, Rühl M, Sauer U. 13C-based metabolic flux analysis. Nat. Protoc. 2009;4(6):878-892. 10.1038/nprot.2009.58 19478804

[17] Young JD. INCA: a computational platform for isotopically non-stationary metabolic flux analysis. Bioinformatics. 2014;30(9):1333-1335. 10.1093/bioinformatics/btu015 PMC3998137 24413674

